# The relationship between parenting stress and parent–child interaction with health outcomes in the youngest patients with type 1 diabetes (0–7 years)

**DOI:** 10.1007/s00431-015-2631-4

**Published:** 2015-10-05

**Authors:** Anke M. Nieuwesteeg, Esther E. Hartman, Henk-Jan Aanstoot, Hedwig J. A. van Bakel, Wilco H. M. Emons, Edgar van Mil, Frans Pouwer

**Affiliations:** Center of Research on Psychology in Somatic diseases (CoRPS), Department of Medical and Clinical Psychology, Tilburg University, Tilburg, The Netherlands; Diabeter, 3011 TG Rotterdam, The Netherlands; Department of TRANZO, Scientific Center for Care and Welfare, Tilburg University, Tilburg, The Netherlands; Department of Methodology and Statistics, Tilburg University, PO Box 90153, 5000 LE Tilburg, The Netherlands; Kidz&Ko, Jeroen Bosch Hospital, ‘s Hertogenbosch, The Netherlands

**Keywords:** Type 1 diabetes mellitus, Parent–child interaction, Behavior, Children, Parents

## Abstract

To test whether parenting stress and the quality of parent–child interaction were associated with glycemic control and quality of life (QoL) in young children (0–7 years) with type 1 diabetes (T1DM), we videotaped 77 families with a young child with T1DM during mealtime (including glucose monitoring and insulin administration). Parent–child interactions were scored with a specifically designed instrument. Questionnaires assessed general and disease-related parenting stress and (diabetes-specific (DS)) QoL. HbA_1c_ (glycemic control) was extracted from the medical records. Both general and disease-related parenting stress were associated with a lower (DS)QoL (r ranged from −0.39 to −0.70, *p* < 0.05), but not with HbA_1c_ levels. Furthermore, with regard to the parent–child interaction, emotional involvement of parents (*r* = 0.23, *p* < 0.05) and expressed discomfort of the child (*r* = 0.23, *p* < 0.05) were related to suboptimal HbA_1c_ levels. There was no clear pattern in the correlations between parent–child interaction and (DS)QoL.

*Conclusion*: The results support the notion that diabetes does not only affect the child with T1DM: T1DM is a family disease, as parenting factors (like stress and parent–child interactions) are associated with important child outcomes. Therefore, it is important for health-care providers to not only focus on the child with T1DM, but also on the family system.

**Table:** 

**What is Known:** *• The incidence of type 1 diabetes is rising, especially in the youngest age group.* *• Research examining the association between parenting factors (like stress and interaction with the child) and child outcomes (like glycemic control and quality of life) in this young patient group is scarce.*
**What is New:** *• Higher levels of parental emotional involvement and more discomfort during disease-specific situations are both related with a less optimal glycemic control in young children with type 1 diabetes.* *• Higher levels of both general and disease-related parenting stress are associated with a lower general and diabetes-specific quality of life of young children with type 1 diabetes.*

## Introduction

Nowadays, the number of children being diagnosed with T1DM is growing, with an overall annual increase of almost 4 % [35]. This number is particularly growing in the youngest age group [3, 35, 50]. In children with T1DM, achieving an optimal HbA_1c_ level (≤58 mmol/mol or 7.5 % [40]) is an important treatment goal. Adequate glycemic control helps to avoid or delay the onset of long-term micro- and macrovascular complications, such as neuropathy, retinopathy, nephropathy and cardiovascular diseases [46]. In addition to these long-term consequences, suboptimal glycemic control is also associated with short-term consequences like a negative effect on school performances [9, 38] and child behavior problems [25, 28]. Despite all efforts, more than half of the children with T1DM still do not reach this optimal HbA_1c_ level [17, 41]. Having T1DM can also have an adverse effect on children’s quality of life (QoL) [49]; therefore, health-care providers not only focus on reaching an optimal HbA_1c_ level, but also on maintaining or enhancing a good QoL in children with T1DM [13]. The literature on QoL in children with T1DM is contradictory as some studies report an obvious impairment in QoL in children with T1DM compared to healthy peers (e.g. [23, 49]), whereas others report a similar level of QoL compared to healthy peers or that children with T1DM even show adaptive outcomes (e.g. [14, 31]). A recent systematic review concluded that the differences in QoL between children with T1DM and healthy peers were, on average, only minimal, although diabetes-specific (DS)QoL problems (e.g. worries, impact on daily functioning) were certainly present [32].

When young children are diagnosed with T1DM, parents have complete responsibility for the daily diabetes management (assessing blood glucose levels, administering insulin, regulating food intake and guarding these parameters in conjunction with the level of physical activity) of their child [10, 42]. Because of these many proceedings, having to think constantly about the correct amount of insulin/carbohydrates, and adjustments during the day without help from daycare or school, parents might consider this as having an ‘extra job’. Therefore, it is not surprising that many parents of young children with T1DM report increased levels of parenting stress [57] and difficulties in parent–child interactions [61]. These parenting factors (parenting stress and parent–child interaction) are strongly linked to parent, child and contextual characteristics [34, 58], like the Process Model of Belsky states [4]. As parent, child and contextual factors are linked to diabetes outcomes [53, 55, 57], parenting stress and parent–child interaction may also be related to the HbA_1c_ level and QoL of the child.

A recent review [8] has described that higher levels of parenting stress were associated with suboptimal HbA_1c_ levels in school-aged children and adolescents (age range 7–17 years). From the same review, it appeared that in younger children (aged 0–11 years), parenting stress was not or even negatively associated with HbA_1c_ levels, indicating that higher levels of stress were related to more optimal HbA_1c_ levels [8]. This discrepancy in findings between parenting stress and HbA_1c_ levels in parents of school-aged children and adolescents versus parents of young children could be due to the fact that parents of older children have a shared responsibility, while parents of younger children have a full responsibility for the care of their children with T1DM. Increased levels of parenting stress could indicate higher levels of involvement in the diabetes regimen and, therefore, more optimal HbA_1c_ levels in young children [8]. The level of parenting stress has not been associated with child QoL often, as the same review [8] only found one study that associated higher levels of parenting stress with a lower child QoL. This study, however, included only children between 12 and 17 years of age [60]. It is important to gain more knowledge about the associations between parenting factors and child outcomes, as parenting stress might be beneficial for HbA_1c_ levels in young children, but not for child QoL [8]. Therefore, knowledge about the association between parenting stress and child QoL in young children with T1DM is insufficient.

Only a few studies have investigated the quality of parent–child interaction in children with T1DM, showing that, particularly, parental over-involvement, parental restrictiveness, parental hostility, conflicts and negative communication are significantly associated with suboptimal HbA_1c_ levels (e.g. [2, 6, 10, 22, 26, 27, 59]) and a lower reported QoL (e.g. [24, 56]). In contrast, positive maternal communication, positive reinforcement, emotional support, parental warmth and caring behavior appeared to be significantly associated with more optimal HbA_1c_ levels (e.g. [22, 27, 62]) and a better reported QoL (e.g. [18, 22]) of children and adolescents with T1DM. However, the aforementioned studies mainly focused on older children (aged 8–20 years) [2, 6, 18, 22, 24, 26, 27, 56, 62] or used rather diverse age groups (aged 4–14) [10, 59], and therefore, research in exclusively (very) young children is limited. We have only found one study that directly observed parent–child interaction in younger children (aged 2–8 years) [37]. This study showed that ineffective parenting during mealtime (i.e., coaxes, interrupted commands and physical prompts) was significantly related to suboptimal HbA_1c_ levels [37]. Currently, research on the parent–child interaction in younger children including also QoL as an outcome factor is lacking.

Research examining the associations among parenting stress, parent–child interaction and child outcomes in young children with T1DM is scarce and urgently needed. More insight in this area may contribute to the development of new, effective interventions. Therefore, the present study was conducted to test whether, and how, parenting stress and the quality of parent–child interaction were related to the HbA_1c_ level and QoL in young children (0–7 years) with T1DM.

## Methods

### Patients and procedure

Children (aged 0–7 years) diagnosed with T1DM more than 6 months and their parents were recruited from 15 hospitals/institutions in the middle and southern part of the Netherlands (including Kidz&Ko, a partnership between seven pediatric diabetes clinics, and Diabeter, a national center for pediatric and adolescent diabetes care and research). Parents who lacked basic proficiency in Dutch and children with an Autism Spectrum Disorder, Down syndrome and/or other mental disabilities (*n* = 17) were excluded. Of the 121 eligible families, 77 families (64 %) agreed to participate. After receiving written informed consent, the mealtime situation (including glucose monitoring and insulin administration) was videotaped during a home visit (for a detailed description of the procedure of the home visits, see Nieuwesteeg et al. [33]). The home visits took place between August 2010 and July 2011. Furthermore, both parents were asked to complete a questionnaire assessing (socio)demographic and clinical variables, general and disease-specific parenting stress and generic and diabetes-specific (DS) child QoL. In the present study, only scores of the questionnaires of the parent who was videotaped during the interaction were used. The study was approved by the Medical Ethical Review board of St. Elisabeth Hospital Tilburg (date: 25-05-2010).

### Measures

#### (Socio)demographic and clinical variables

The first part of the questionnaire included items involving (socio)demographic characteristics (i.e. gender and age of the child, marital status and educational levels of both parents) and clinical characteristics (i.e. treatment regimen, frequency of blood glucose monitoring (average number of assessments/day) and years since diagnosis).

#### Metabolic control

Glycemic control (HbA_1c_ level) was determined at the hospital where the child was treated for T1DM and extracted from the medical records of the children, after receiving written consent from the parents. In clinical care, the HbA_1c_ level is generally measured once every 2–3 months and gives an impression of the average blood glucose level over a 6- to 12-week period. Using the patient’s charts, we included the HbA_1c_ level measured closest to the home visit (maximum of 3 months before or after the home visit).

#### Parent–child interaction

To assess the quality of parent–child interaction, the mealtime situation (including glucose monitoring and insulin administration) was videotaped in all participating families during a home visit. The videotapes were scored by an observer (AN) with the qualitative OKI-DO observation instrument, which is specifically developed for children with T1DM, to assess the quality of parent–child interaction during diabetes-specific situations [33]. This observation instrument comprises ten domains, including four parent domains (‛emotional involvementʼ, ‛limit settingʼ, ‛respect for autonomyʼ and ‛quality of instructionʼ), four child domains (‛negative behaviorʼ, ‛avoidanceʼ, ‛cooperative behaviorʼ and ‛child’s response to injectionʼ) and two family domains (‛emphasis on diabetesʼ and ‛mealtime structureʼ). The parent–child dyad will receive a score (1–5) on each domain, in which higher scores reflect more of the behavior (e.g. a high score on ‘emotional involvement’ means the parent is highly emotional involved, and a high score on ‘negative behavior’ means the child shows a lot of negative behavior). An example of the rating scale ‛respect for autonomyʼ can be rated varying from score 1:

‘The caregiver receives this score if he/she fully determines what should happen without explaining anything to the child and with a visible lack of respect for the autonomy. For example, the caregiver just takes the finger of the child to check the glucose, (harshly) ‘pulls’ the child in the correct position to operate the insulin pump or determines (without consulting or warning the child) where and when the insulin injection takes place, the caregiver fully determines what and how much the child eats. If the child is (rather) independent in managing his/her diabetes, the caregiver receives this score if he/she repeatedly interferes when the child is managing his/her diabetes, while it is clear from the observation that the child can perform everything on its own’.

To score 5: ‘The caregiver receives this score if he/she praises initiatives of the child and encourages the child to make decisions on his own regarding his/her diabetes. The child may, for example, read the glucose meter, operate the insulin pump or determine where and when the insulin injection takes place (the caregiver could of course check the things his/her child does, but is herein not at all intrusive). Everything is determined in consultation with the child and the child is treated with respect.’

Research shows encouraging indications for the usability, reliability and preliminary validity of the OKI-DO instrument to assess parent–child interaction in young children with T1DM during mealtime (including glucose monitoring and insulin administration) [33].

#### General parenting stress

General parenting stress was assessed with the short Dutch version of the Parenting Stress Index [1, 12]. This is a 17-item self-report measure, in which parents report how much they agree on the propositions about stress in the parent–child system. The items are rated from 1 (strongly disagree) to score 4 (strongly agree). All items were summed to get a total score (*α* = 0.92). Higher scores reflect more general parenting stress. The 17-item version has shown good reliability [48].

#### Disease-related parenting stress

Disease-related parenting stress was assessed with a validated, 42-item self-report measure, the Pediatric Inventory for Parents (PIP) [44, 54]. Parents are asked to describe both the frequency and difficulty of experienced disease-related parenting stress across four domains: ‛communicationʼ (for example: with the medical team, partner, child), ‛emotional distressʼ (for example: quality of sleep, effect on mood), ‛medical careʼ (for example: treatment demands) and ‛role functioningʼ (for example: being able to go to work). All items are scored on a 5-point Likert scale on both *frequency* and *difficulty*. The scale scores were summed to get a total *frequency* (*α* = 0.93) and total *difficulty* score (*α* = 0.93). Higher scores reflect more frequency and difficulty in disease-related parenting stress. Adequate internal consistency and construct validity of the original version of the PIP have been reported [44].

#### QoL of the children

Generic QoL was measured with the TNO-AZL Preschool Quality Of Life questionnaire or TAPQOL [15] in children in the age of 1 through 5 years of age and the TNO-AZL Child Quality Of Life questionnaire or TACQOL [52] in children of 6 and 7 years of age. These multidimensional questionnaires are proxy measures, as they assess the parent’s perceptions of health-related QoL in (preschool) children. The TAPQOL includes 43 items constituting 12 scales covering aspects of QoL: seven health-related scales: stomach problems, skin problems, lung problems, sleeping problems, appetite, motor functioning and communication (these seven scales were summed and then averaged (health-related QoL), *α* = 0.80) and five psychosocial-related scales: liveliness, positive mood, problem behavior, anxiety and social functioning (these five scales were summed and then averaged (psychosocial QoL), *α* = 0.75). The TACQOL includes 63 items constituting seven scales covering aspects of QoL: five health-related scales: pain and symptoms, motor function, autonomy, cognitive functioning and interaction with parents and peers (these five scales were summed and then averaged (health-related QoL), *α* = 0.94), and two psychosocial-related scales: experience of positive and negative emotions (the two scales were summed and then averaged (psychosocial QoL), *α* = 0.84). In both questionnaires, the parent indicates to what extent specific problems of the health-related functioning scales occurred in the past few weeks, with three response categories: ‘never’, ‘sometimes’ and ‘often’. If a problem occurs, the parents are asked how the child is feeling: ‘(very) good’, ‘not so good’, ‘pretty bad’, and ‘bad’. For each item, the two answers are combined into a single item score ranging from 0 to 4 (‘never’ 4 and ‘sometimes’ or ‘often’ combined with ‘(very) good’ 3, ‘not so good’ 2, ‘pretty bad’ 1, and ‘bad’ 0). With the psychosocial-related scales, the parents indicate on a Likert scale whether a certain emotion in their child has appeared in the last few weeks (never, sometimes, often). Item scores for the psychosocial-related scales run from 0 to 2. All TAPQOL and TACQOL scales are linearly transformed to 0–100 scales; higher scores will correspond to a better QoL. Research showed that the TAPQOL and the TACQOL are reliable and valid questionnaires [16, 19].

Diabetes-specific QoL (DSQoL) was measured with a child self-report questionnaire (Smiley Faces) for young children composed by the Hvidøre Study Group [21]. This questionnaire has been modified in a proxy-report form with permission of the authors so that the parents can complete the questionnaire for their child. The questionnaire comprises 19 items about feelings of the child in relation with his or her diabetes (for example, items about administering insulin, health, leisure time, and school/nursery/daycare). The items are rated on a 5-point Likert scale. Scores are (re)coded so that a higher score corresponds to a better QoL. All items were summed to get a total diabetes-specific QoL score (*α* = 0.86). The originally child self-report questionnaire showed good reliability and validity [21].

#### Statistical analyses

Statistical analyses were conducted using the Statistical Package for the Social Sciences (SPSS, version 19). Frequencies and descriptive statistics were used to present the (socio)demographic and diabetes-related characteristics of the participating families. Pearson’s correlation coefficients were calculated to examine the strength of the relationships between parenting stress and the parent–child interaction with HbA_1c_ levels and (DS)QoL. Because different QoL questionnaires were used for children aged 0–5 and 6–7, we have examined the Pearson’s correlations for these questionnaires (generic and DS QoL) separately. The results of the correlations were considered statistically significant when *p* ≤ 0.05. Furthermore, the effect sizes were examined. According to Cohen, *r* of 0.10, 0.30 and 0.50 are considered as small, medium and large effects, respectively [7]. We did not perform a power analysis.

## Results

### Sociodemographic and clinical data

Table 1 summarizes the characteristics of participating parents and children. Among the participating children, there were 41 boys (53 %). The children with T1DM had a mean age of 5 years (SD = 1.5, range: 2–7 years). Most children (82 %) received pump therapy. On average, parents monitored their child’s blood glucose 6 times a day (range: 2–20). The mean HbA_1c_ level of the children, measured closest to the home visit, was 59 mmol/mol or 7.6 % (range 32–80 mmol/mol or 5.1–9.5 %).

**Table 1:** (Socio)demographic and clinical characteristics of young children with type 1 diabetes and their parents

			Number (%)	Median (percentile 25–75)	M	SD
Children	Sex	Boys	41 (53 %)			
		Girls	36 (47 %)			
	Age (years)	(range 2–7)		5 (4–6)		
	HbA_1c_	(range 32 mmol/mol-80 mmol/mol)	77		59	9.03
		(range 5.1 %–9.5 %)	77		7.6 %	0.8 %
	Treatment	Insulin pump	63 (82 %)			
		Multiple daily insulin injections	14 (18 %)			
	Blood glucose monitoring	Times a day (range 2–20)		6 (5–7)		
	Years since diagnose	(range 1–6 years)		2 (1–3)		
			N (number per total)			
Parents	Total	Mothers observed	74 (74/77)			
		Fathers observed	3 (3/77)			
	Marital status (mothers)	Single	5 (5/74)			
		Cohabiting	10 (10/74)			
		Married / registered partners	52 (52/74)			
		Missing	7 (7/74)			
	Marital status (fathers)	Single	0 (0/3)			
		Cohabiting	1 (1/3)			
		Married / registered partners	2 (2/3)			
		Missing	0 (0/3)			
	Educational level (mothers)	Primary education	1 (1/74)			
		About 12 years of formal education	36 (36/74)			
		15–16 years of formal education	29 (29/74)			
		Other	1 (1/74)			
		Missing	7 (7/74)			
	Educational level (fathers)	Primary education	0 (0/3)			
		About 12 years of formal education	0 (0/3)			
		15–16 years of formal education	3 (3/3)			
		Other	0 (0/3)			
		Missing	0 (0/3)			

Of the 77 observed parents (96 % mothers), 70 parents (96 % mothers) completed the questionnaire about general parenting stress and DSQoL of their child, 68 parents (96 % mothers) completed the questionnaire about the generic QoL of their child and 64 parents (94 % mothers) completed the questionnaire about disease-related parenting stress. Most of the 70 parents (96 % mothers) who completed the questionnaires were cohabiting or married/registered partners (7 % of the mothers and 0 % of the fathers were single). More than half of the participating mothers (54 %) had a higher educational level (i.e. approximately 12 years of formal education), while all fathers (100 %) had a Bachelor’s or Master’s degree (i.e. approximately 15 years of formal education).

### Inter-rater reliability of the observations

To assess the inter-rater reliability of the OKI-DO observations, a second observer (HvB) scored 20 % of the videotapes independently of the first observer (AN). The agreement with the first observer was high as weighted kappa was 0.73, indicating a good inter-rater reliability.

### Associations between parenting stress and the quality of parent–child interaction with HbA_1c_ levels and (diabetes-specific) quality of life

Table 2 summarizes the Pearson correlations between general parenting stress, disease-related parenting stress and the domains of the quality of parent–child interaction with the HbA_1c_ level and (DS)QoL of the children.

**Table 2:** Relationships between (disease-related) parenting stress, the OKI-DO domains and child (health) outcomes

		0–5 years			6–7 years		
	HbA_1c_	Generic QoL		DSQoL	Generic QoL		DSQoL
		Health related	Psycho-social	Total	Health related	Psycho-social	Total
	*r*	*r*	*r*	*r*	*r*	*r*	*r*
Parenting stress							
General total	0.12	−0.13	−0.18	−0.42**	−0.53**	−0.56**	−0.70**
Disease-related frequency total	−0.15	−0.19	−0.18	−0.39*	−0.56**	−0.40*	−0.69**
Disease-related difficulty total	−0.09	−0.16	−0.10	−0.33	−0.52*	−0.27	−0.70**
Parent–child interaction							
Emotional involvement	0.23*	−0.12	0.13	−0.04	0.02	−0.01	0.25
Limit setting	0.08	−0.17	0.07	0.03	−0.13	−0.12	0.02
Respect for autonomy	−0.05	−0.17	−0.11	−0.22	0.08	0.19	0.23
Quality of instruction	−0.12	−0.16	−0.08	0.02	0.13	0.41*	0.19
Negative behavior	0.03	0.20	0.08	0.26	0.35	0.22	0.15
Avoidance	−0.02	0.15	0.16	0.08	−0.06	−0.30	−0.16
Cooperative behavior	0.11	−0.12	0.03	−0.02	0.18	−0.05	0.16
Child’s response to injection	0.23*	−0.16	−0.01	−0.14	0.03	−0.32	−0.13
Emphasis on diabetes	0.14	0.07	0.22	0.36*	0.24	0.08	−0.10
Mealtime structure	−0.02	−0.19	−0.10	−0.11	−0.25	−0.19	−0.07

*p < .05; **p < .01

Results showed that general and disease-related parenting stress were significantly negatively associated with both generic QoL (correlations ranging from −0.40 to −0.56, medium to large effect sizes) and diabetes-specific QoL (correlations ranging from −0.39 to −0.70, medium to large effect sizes). HbA_1c_ level did not correlate significantly with general or disease-related parenting stress. These results indicate that elevated levels of both general and disease-related parenting stress were associated with lower (DS)QoL of young children with T1DM.

Furthermore, only the OKI-DO domains *emotional involvement* (*r* = 0.23, small effect size) and *child’s response to injection* (*r* = 0.23, small effect size) were significantly positively associated with the Hba_1c_ level of the child, which indicates that parents with children with higher HbA_1c_ levels were more emotionally involved and that children with higher HbA_1c_ levels expressed more discomfort during glucose monitoring (for example, more tension in body, tightening eyes, crying or resisting behavior). A better *quality of instruction* was associated with a better generic QoL in children aged 6–7 (*r* = 0.41, medium effect size), and more *emphasis on diabetes* during the meal was significantly correlated with a better DSQoL in children aged 0–5 (*r* = 0.36, medium effect size).

## Discussion

For health-care providers, maintaining or enhancing a good QoL of children with T1DM is as important as guarding or achieving good glycemic control [13]. However, research examining factors that are associated with HbA_1c_ levels and (DS)QoL in young children with T1DM is still scarce. The present study focused on this youngest patient group and found that higher levels of (disease-related) parenting stress were associated with a lower (DS)QoL of the child and that emotional involvement and the child’s response to injection were associated with lower HbA_1c_ levels.

The results showed that parenting stress was not associated with HbA_1c_ levels. A previous study, however, concluded that higher levels of disease-related parenting stress were associated with more optimal HbA_1c_ levels in young children with T1DM [7]. An explanation of the discrepancy in findings between our study (no significant correlation) and the study of Stallwood [43] might be explained by type of insulin treatment: parents generally experience less disease-related parenting stress when their child is on insulin pump therapy [29, 45]. In the study of Stallwood [43], none of the children used pump therapy, in contrast to 84 % of the children in the present study. Future research should examine the association between glycemic control and disease-related parenting stress in a sample with a comparable amount of children using multiple daily insulin injections versus insulin pump therapy to see whether there is or is not a significant association between disease-related parenting stress and HbA_1c_ levels. If there indeed is a significant association, appropriate interventions in order to lower disease-related parenting stress could be part of the treatment in lowering HbA_1c_ levels.

Furthermore, parents who experienced higher levels of (disease-related) parenting stress reported lower child QoL, especially DSQoL (e.g. problems with insulin administration, glucose monitoring, energy level, health, etc.). This is in line with a study examining the associations between parenting stress and the QoL of teenagers with T1DM [60], but the present study is the first study that has examined this relationship in young children with T1DM. Because of these results, health-care providers should not only be aware of the (DS)QoL of the child, but also the perceived (disease-related) parenting stress of the parents as higher levels of (disease-related) parenting stress are associated with lower (DS)QoL. Therefore, health-care providers should monitor the level of (disease-related) parenting stress regularly in order to avoid a low QoL of the child.

Parents were more emotionally involved when their child had a suboptimal HbA_1c_ level. Research with older children and adolescents with T1DM also showed that a suboptimal HbA_1c_ level was positively associated with emotional involvement of the parents [26] and involvement in diabetes care [6]. However, the causal direction of this association remains unclear. It could be that parents are more emotionally involved when trying to get a more optimal HbA_1c_ level and thereby delaying the onset of long- and short-term complications [26]. Otherwise, it could also be true that parents who are highly emotionally involved during diabetes-specific situations deliberately set higher HbA_1c_ targets for their child because of fear of hypoglycemia [36].

Furthermore, the results showed that children with a high HbA_1c_ level expressed more discomfort during glucose monitoring and/or insulin administration (for example, more tension in body, tightening eyes, crying or resisting). Displaying more discomfort could be due to some form of needle phobia or a low pain threshold. When children experience needle phobia or have a low pain threshold, parents might postpone or omit insulin injections or glucose monitoring in order to avoid these stressful and unpleasant situations, which could result in suboptimal HbA_1c_ levels [20]. It could also be true that a high HbA_1c_ level might ‘worsen’ the diabetes for both parent and child, and that the child, due to an emphasis on their disease, experiences more discomfort during glucose monitoring and/or insulin administration.

Remarkably, the other domains of parent–child interaction around the diabetes situation were not related to HbA_1c_ level. Based on results of a previous study with young children [37] and studies with older children [2, 6, 10, 22, 26, 27, 59], showing that ineffective parenting strategies and child misbehavior were significantly correlated with less optimal HbA_1c_ levels, we expected that, for example, less *respect for autonomy* or more *negative behavior* of the child would be associated with suboptimal HbA_1c_ levels. This discrepancy in findings might be due to different sample characteristics, as our sample had more optimal HbA_1c_ levels (7.6 % or about 59 mmol/mol in our study versus >8 % or >64 mmol/mol [2, 6, 10, 26, 37]). Furthermore, the children in our sample were much younger than in most other studies [2, 6, 10, 22, 26, 27, 59]. Also, it is possible that families who encountered problems (like child misbehavior) during the mealtime situation (including glucose monitoring and insulin administration) were reluctant to participate in the present study. Our sample consisted of rather high-functioning families, meaning that they did not encounter major problems during mealtime, glucose monitoring and insulin administration [33].

The quality of parent–child interaction did not show clear patterns of correlations with (DS)QoL. Previous research with youth with T1DM showed that parent–child behaviors (e.g. diabetes-specific family conflict, warmth) were related to QoL [18, 24]. The parent–child behaviors in these studies were collected through self-report questionnaires [18, 24]. Weisberg-Benchell [56] also examined the relationship between QoL and parent–child behavior, but they used both self-report questionnaires and observations to assess parent–child behaviors. While the self-reported parent–child behaviors did correlate with QoL, the observed parent–child behaviors did not [56]. This highlights the importance of using direct observations in examining the quality of parent–child interaction in future studies, with an observational instrument like the OKI-DO instrument, as self-reports might reflect a more subjective view.

The present study has some limitations that need to be described. Research has shown that diabetes centers not only differ in structural issues, but also at an educational level and guidance regarding diabetes, which could influence glycemic control [11]. Because we included children from 15 different hospitals/institutions, the diabetes education and guidance these parents and children receive(d) could be very different. Furthermore, our sample consisted of almost only Caucasian participants (97 %). Participation rate among fathers was very low, and most of the parents had a relatively high educational level, which is not a fair representation of the population in the Netherlands [51]. As the educational level and socioeconomic status of parents are positively associated with the quality of parent–child interaction [47] and parenting strategies [5, 39], the results of the present study may not be generalized to parents with a lower educational level. Also, because of the videotaped home visits, it is possible that families who frequently experience problems during mealtime, glucose monitoring and/or insulin administration were reluctant to participate in our study, which might have led to biased results. Future research, therefore, should examine whether a causal relationship exists between the quality of the parent–child interaction and (disease-related) parenting stress with (health) outcomes of children with T1DM, including families of different ethnic backgrounds, parents with lower educational levels and families who encounter problems during diabetes-specific situations.

A strength of the present study is the use of the OKI-DO instrument, as observations of “real-life” parent–child interactions and scored by an independent observer can provide more objective data to assess the quality of parent–child interaction than using self-report questionnaires or interviews [33]. The OKI-DO instrument could be used by health-care providers to evaluate interventions (like video interaction guidance) aimed at optimizing the quality of parent–child interaction and/or lowering the parenting stress in order the influence child outcomes. Furthermore, as recommended by Nakagawa [30], we refrained from correcting for examining multiple associations to be able to give an overview of all the associations that were examined as we were interested in all possible associations between the included variables. Another strength is the support from several large diabetes clinics in the Netherlands. Therefore, it was possible to focus on younger children with T1DM and their families, which makes this study innovative as this patient group is understudied.

The results of the present paper support the notion that diabetes does not only affect the child with T1DM: T1DM is a family disease, as parenting factors (like stress and parent–child interactions) are associated with important child outcomes. Therefore, it is important for health-care providers to not only focus on the child with T1DM, but also on the family system.

## Abbreviations

DSQoL: Diabetes-specific quality of life
HbA_1c_: Hemoglobin A_1c_ or glycosylated hemoglobin A protein
QoL: Quality of life
T1DM: Type 1 diabetes mellitus
